# Case Report: EBV-Positive Extra-Nodal Marginal Zone Lymphoma Associated With XMEN Disease Caused by a Novel Hemizygous Mutation in *MAGT1*

**DOI:** 10.3389/fonc.2021.653266

**Published:** 2021-03-18

**Authors:** Xin Huang, Dan Liu, Zifen Gao, Cuiling Liu

**Affiliations:** ^1^ Department of Pathology, School of Basic Medical Science & Third Hospital, Peking University Health Science Center, Beijing, China; ^2^ Department of Pathology, Children’s Hospital, Capital Institute of Pediatrics, Beijing, China

**Keywords:** EBV, XMEN disease, immunodeficiency, MAGT1 gene, extranodal marginal B cell lymphoma

## Abstract

**Background:**

X-linked immunodeficiency with magnesium defect and Epstein-Barr virus infection and neoplasia (XMEN) disease is an X-linked genetic disorder of immune system caused by loss-of-function mutation in gene encoding Magnesium transporter 1 (MAGT1). Individuals with XMEN disease are prone to developing Epstein Barr Virus (EBV)-associated lymphomas. Herein, we report the first known case of an EBV+ EMZL associated with XMEN disease.

**Case presentation:**

The patient was an 8-year-old Chinese boy who suffered from recurrent infections from birth. Six months before, the patient presented with a painless mass on his upper lip and excisional biopsy revealed an EBV-positive extra-nodal marginal zone lymphoma (EBV+ EMZL). Furthermore, molecular investigations with next-generation sequencing identified a novel germline mutation in MAGT1 (c.828_829insAT) in the patient. The c.828_829insAT variant was predicted to cause premature truncation of MAGT1 (p.A277M.fs*11) and consequently was defined as likely pathogenic. The mutation was inherited from his asymptomatic heterozygous carrier mother. Hence the patient was diagnosed with an XMEN disease both clinically and genetically.

**Conclusion:**

Our results expand the genetic spectrum of XMEN disease and also the clinical spectrum of EBV+ EMZL. We highlight the importance of the genetic etiology underlying EBV+ lymphoma in the pediatric population.

## Introduction

Epstein-Barr virus (EBV)-positive extra-nodal marginal zone lymphoma (EMZL) has been recognized as a rare type of monomorphic post-transplantation lymphoproliferative disorders in the current 2017 World Health Organization lymphoma classification (1). More recent studies broadened the spectrum of EBV+ EMZL not only in the post-transplant patients but also in various immunodeficiency settings (2).

X-linked immunodeficiency with magnesium defect and Epstein-Barr virus infection and neoplasia (XMEN) disease belongs to the category of X-linked combined primary immunodeficiency caused by mutation in the gene Magnesium transporter 1 (*MAGT1*) (3–5). *MAGT1* encodes a highly selective transporter for Mg(2+) that acts as a crucial regulator for basal intracellular free magnesium levels (6). MAGT1 is evolutionarily conserved and ubiquitously expressed. It plays an essential role in cellular growth and survival. More recently, a crucial role for MAGT1 in the regulation of T- and NK-cell function has been well-established (3, 5, 7), serving as a potential mechanism underlying the immunodeficiency of XMEN disease. The common presenting features of XMEN disease are recurrent infections involving respiratory tract, idiopathic CD4+ T-cell lymphopenia, and susceptibility to EBV+ lymphomas (3, 4). Importantly, EBV-positive lymphomas remain a major cause of morbidity. Herein, we describe a case of EBV-positive EMZL in an 8-year-old Chinese boy with genetically confirmed XMEN disease and a novel germline mutation in *MAGT1* gene (NM_032121.5: c.828_829insAT, p.A277M.fs*11) as a cause.

## Case Description

The patient is an 8-year-old Chinese boy born as the sole child of unrelated parents. In his medical history, the patient experienced recurrent upper and/or lower respiratory tract infection 3-4 times per year from birth. 6 months before, the patient presented with a painless mass measuring 2 x 2 cm on his upper lip. He had no lymphadenopathy and/or organomegaly. HIV antibody was negative and the complete blood count was within normal range. Absolute lymphocyte count revealed a decreased CD4+ T-cell count of ~1075 cells/μl with a CD4 to CD8 ratio of 0.7. Additionally, the patient was detected with an elevated EBV-DNA copy number (5.5*10^3 copies/ml).

**Figure 1:**
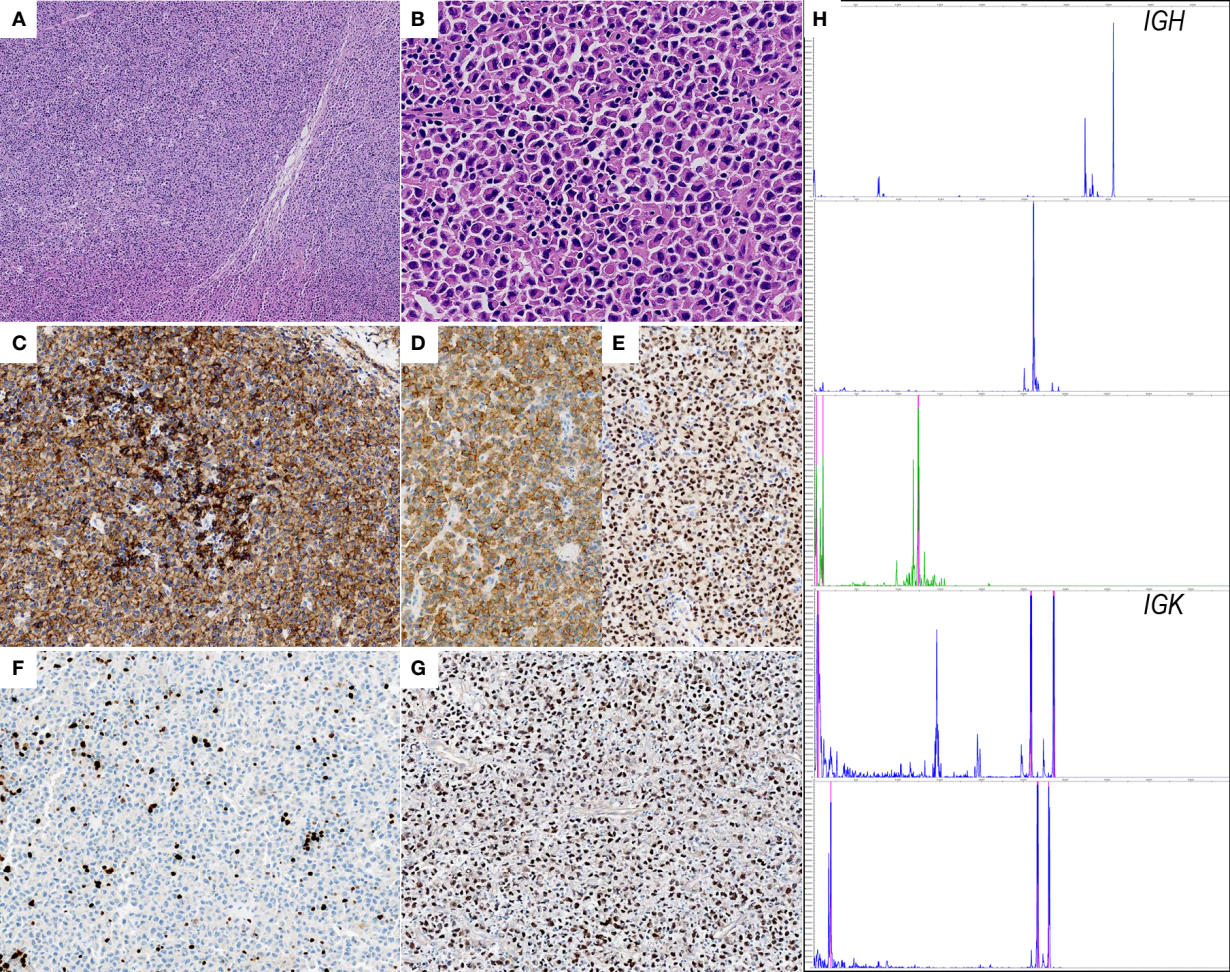
Histopathological features of EBV+ EMZL. **(A)** Hematoxylin and eosin staining of the excisional tumor biopsy displays dense infiltrate of small to medium-sized lymphoplasmacytic cells with a vaguely nodular arrangement; **(B)** the higher magnification shows classic plasmacytoid cytomorphology with Dutcher bodies; **(C)** CD20 is positive in neoplastic cells. Note that the CD20 staining intensity is clearly lower in plasmacytoid cells than in lymphoid cells; **(D, E)** CD38 and MUM1 are strongly positive confirming the plasmacytic differentiation; **(F)** Ki-67 immunostaining demonstrates scattered positivity in the tumor cells, indicating low proliferative activity; **(G)** the neoplastic cells are uniformly and strongly positive for EBER. **(H)** Electropherograms reveal monoclonal peaks in *IGH* and *IGK* framework regions.

Excisional biopsy revealed dense infiltrate of small to medium-sized lymphoplasmacytic cells imparting a vaguely nodular arrangement at low power (**Figure 1A**). Under high-power, 2 types of neoplastic cells were present, 1) small round to oval cells with scant pale cytoplasm and loosely clumped chromatin, 2) cells exhibited plasmacytoid appearance with Dutcher bodies (**Figure 1B**). Immunostaining with a panel of antibodies was performed. Results showed that the neoplastic cells were diffusely immunoreactive for pan-B cell markers (CD79a, CD20, and CD19) with a lower staining intensity in the fraction of plasmacytoid cells (**Figure 1C**). As expected, the plasmacytoid cells were positive for plasma cell markers (CD38 and MUM1) (**Figures 1D, E**). Ki-67 proliferation index was estimated to be 5-10%, consistent with the indolent feature of this entity (**Figure 1F**). Additionally, the EBER testing demonstrated a strong and diffuse immunoreactivity in the neoplastic cells (**Figure 1G**). Further PCR-based clonal analysis disclosed clonal immunoglobulin (IG) gene rearrangement (**Figure 1H**). The morphologic and the immunophenotypic features together with the EBV status and the clonal assessment were consistent with EBV-positive EMZL.

**Figure 2:**
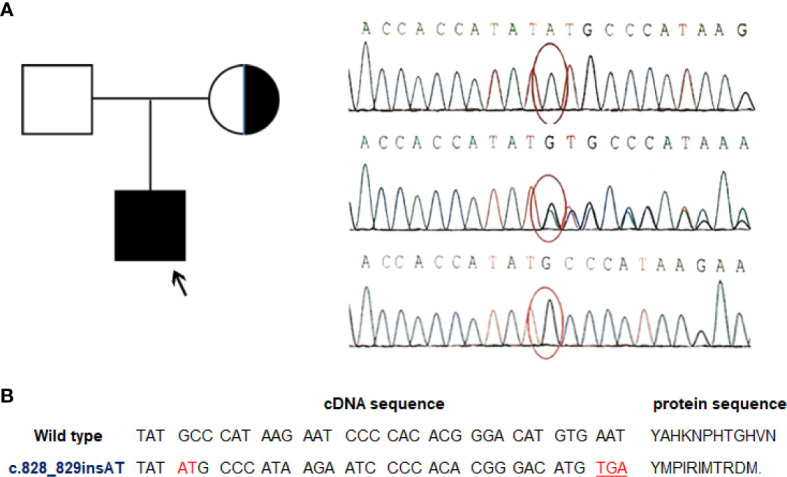
A novel *MAGT1* mutation identified in the current patient. **(A)** Family pedigree and DNA sequence chromatograms of the affected child and his parents. The proband was indicated by arrow. Confirmations of the in-frame insert mutation in *MAGT1* by Sanger sequencing in the proband (top), his mother (middle), and his father (bottom). Nucleotide mutation site is marked with red circle. Reference sequence for *MAGT1* gene: NM_032121.5 **(B)** Overview of the identified mutation. The c.828_829insAT mutant leads to a premature termination codon and generates a truncated MAGT1 protein (p.A277M.fs*11).

EBV+ EMZL is referred to occur only in immunocompromised individuals so far. For the present patient, his medical history illustrated that he seemed vulnerable to respiratory infections from birth. Besides, the laboratory test results disclosed an unexplained CD4+ T-cell lymphopenia. The finding of EBV+ EMZL along with CD4+ T-cell lymphopenia in a child with a clinical history of recurrent infections prompted us to suspect certain immunodeficiency in the patient contributing to his illness. To elucidate the potential genetic causes, a thorough screening for candidate genes using targeted next generation sequencing (NGS) analysis of a set of immune-related genes was performed with genomic DNA obtained from the patient and his parents. Not surprisingly, the analyses revealed an in-frame insert mutation in *MAGT1* gene (c.828_829insAT) in the patient. Subsequently, the mutation was validated by conventional Sanger sequencing assay. Meanwhile, genetic testing of his parents indicated that the mutation was inherited from his healthy mother who was a hemizygous carrier of this variant whereas his father was wild-type (**Figure 2A**). This *MAGT1* variant has not been reported previously. It is located in *MAGT1* exon 6 with 2-nucleotide insertion between the position 828 and 829 (c.828_829insAT). As a result, it changes the alanine at codon 277 to methionine and introduces 10 erroneous amino acids followed by a premature stop codon TGA at codon 287 (p.A277M.fs*11) (**Figure 2B**). As a result, it leads to the production of a frameshift truncated MAGT1 protein of 287 amino acids (p.A277M.fs*11), while the wild-type protein consists of 367 amino acids. According to the American college of Medical Genetics and Genomics standard and guidelines, this frameshift variant is predicted to be likely pathogenic. Collectively, the patient fulfills the diagnostic criteria of XMEN disease in both clinical and genetic aspects.

The combined data from clinical evaluations, pathologic findings, and genetic testing suggested an XMEN disease and the resultant development of EBV+ EMZL. The patient has received 4 cycles of Rituximab in combination with CHOP chemotherapy for the lymphoma and he responds well.

## Discussion

A recent study by Gong S et al. (2) identified that EBV+ EMZL can occur in diverse immune defects, but primary immunodeficiency is very uncommon. Until now, only 2 documented cases were associated with primary immunodeficiency, including one with *RMRP* mutation and the other with ataxia telangiectasia (2). We here report an additional case of EBV+ EMZL associated with another form of primary immunodeficiency, XMEN disease. A total of 10 EBV+ lymphomas associated with XMEN disease were described, including 6 cases of classical Hodgkin lymphoma, 3 cases of diffuse large B-cell lymphoma, and 2 cases of Burkitt lymphoma (3–5, 8, 9). The present case of EBV+ EMZL expands the spectrum of EBV+ lymphomas in patients with XMEN disease.

Mutation in the gene *MAGT1* is proposed as the genetic defect underlying XMEN disease. Diagnosis of XMEN disease is based on the combination of genetic and clinical findings. Our patient’s manifestations (recurrent infections from birth, CD4+ T-cell lymphopenia, and the development of EBV+ lymphoma) were highly suggestive of certain primary immunodeficiency disorder (3, 4). Subsequent NGS-based genetic analysis revealed a frameshift mutation of *MAGT1* (c.828_829insAT) in the patient. Moreover, this variant was inherited from his asymptomatic mother who was confirmed as a heterozygous carrier. The c.828_829insAT variant is previously unreported that is assumed to result in an altered protein sequence and a truncated protein (p.A277M.fs*11), supporting the deleterious nature of this novel mutation and the diagnosis of XMEN disease.

In summary, we present here the first case of EBV+ EMZL associated with XMEN disease and a novel frameshift mutation c.828_829insAT in *MAGT1* as a cause. This case expands the mutational spectrum of XMEN disease and also the clinical spectrum of EBV+ EMZL. Our findings emphasize the genetic defects in pediatric patients with EBV+ lymphomas. Additionally, we highlight that appropriate use of NGS technologies has many benefits for an early and accurate diagnosis of genetic disorders.

## Data Availability Statement

The datasets presented in this article are not readily available as requested by the patient and his family to protect his privacy. Requests to access the datasets should be directed to the corresponding author.

## Ethics Statement

Written informed consent was obtained from the minor(s)’ legal guardian/next of kin for the publication of any potentially identifiable images or data included in this article.

## Author Contributions

XH, CL and ZG were responsible for scoring the immunohistochemistry, EBER, and the clonality analysis. DL contributed materials and data collection. XH, CL and ZG wrote the paper. All authors contributed to the article and approved the submitted version.

## Conflict of Interest

The authors declare that the research was conducted in the absence of any commercial or financial relationships that could be construed as a potential conflict of interest.

## Abbreviations

EBV, Epstein Barr Virus; EMZL, Extranodal Marginal Zone Lymphoma; XMEN disease, X-linked immunodeficiency with magnesium defect and Epstein-Barr virus infection and neoplasia; MAGT1, Magnesium transporter 1.
